# Roots Structure and Development of *Austrobaileya scandens* (Austrobaileyaceae) and Implications for Their Evolution in Angiosperms

**DOI:** 10.3390/plants9010054

**Published:** 2020-01-01

**Authors:** Julien B. Bachelier, Imran Razik, Maria Schauer, James L. Seago

**Affiliations:** 1 Structural and Functional Plant Diversity Group, Institute of Biology, Freie Universität Berlin, Altensteinstrasse 6, 14195 Berlin, Germany; 2 Department of Biological Sciences of State University of New York (SUNY) at Oswego, 30 Centennial Drive, Oswego, NY 13126, USA; 3 Seago Botanical Consulting, Minetto, NY 13115, USA

**Keywords:** ANA grade, Austrobaileyales, common initials, magnoliids, open meristem

## Abstract

Since the resolution of the ANA grade [Amborellales, Nymphaeales, Austrobaileyales] as sister to all other flowering plants, a few comparative studies of root structure have suggested that some of their anatomical traits could be of importance to understanding root evolutionary development and angiosperm phylogeny. However, there is still a paucity of information on root structure and apical meristems (RAMs) in these lineages and especially the sister to all other Austrobaileyales, *Austrobaileya scandens*. We used microtome sections and bright field, epifluorescence, laser confocal, and scanning electron microscopy to study adventitious root RAMs and tissues of *A. scandens*. Our results indicate that root structure is relatively simple in *A. scandens*. The epidermis has a thick cuticle and lacks root hairs. The stele is typically diarch, or some modification thereof, and surrounded by a cortex differentiated into a uniseriate endodermis, a middle region sometimes packed with starch, some oil cells, and colonized by arbuscular mycorrhizal fungi, and a multiseriate exodermis. Secondary growth produced many vessel elements in the secondary xylem and scattered sclerenchymatous fibers in secondary phloem. The absence of distinct patterning within the RAM and between the RAM and derivative differentiating tissues shows that the RAM is open and characterized by common initials. Roots structure and anatomy of *A. scandens* are thus essentially similar to some previously described in *Amborella* or *Illicium* in the ANA grade and many magnoliids, and suggest that the first woody flowering plants likely had an open RAM with common initials. Their functional and evolutionary significance in woody early-diverging and basal lineages of flowering plants and gymnosperms remains unclear, but they are clearly ancestral traits.

## 1. Introduction

Since the resolution of the ANA grade [Amborellales, Nymphaeales, Austrobaileyales] as sister to all other flowering plants, most studies of anatomical traits that might be relevant to understanding the evolutionary development and phylogeny of angiosperms have been limited to multiple floral reproductive characters and to certain leaf and xylem traits [1,2]. However, it has been suggested that certain root traits might also be important to elucidate their elusive origin and rapid diversification [3,4,5].

One significant root character is the occurrence of common initials, or CI, in root apical meristems, or RAM. In CIs, there is no clear and distinct boundary among the meristematic cells and tissues at the base of the root cap and the tips of the main root tissues, protoderm, ground meristem, and procambium, in the open RAMs of Amborellales and other woody species of the ANA grade (Austrobaileyales) and magnoliids (Laurales, Magnoliales, Canellales) [4]. Furthermore, the RAMs of basal angiosperms turn out to be very similar to those in many gymnosperms, which are also known for having common initials for most tissues of the root apex [6,7,8]. Indeed, Heimsch and Seago [4] noted that gymnosperm CI RAMs, as reported by Pillai [8], are also similar, when present, to those found in the more variable RAMs of Amborellales and Schisandraceae of the Austrobaileyales. However, with the exception of *Illicium*, there is relatively little or no observation on the RAM and root developmental anatomy of other members of the Schisandraceae or Austrobaileyales, despite its position as sister to all other members of the order. *Austrobaileya scandens* roots have received little attention. Bailey and Swamy [9] studied *A. scandens* but not its roots. Metcalfe and Chalk [10] included *A. scandens* as a member of the Schisandraceae and reported very little on it and nothing on its roots. Dickison and Endress [11], Baranova [12,13], Feild et al. [14], and Carpenter [15,16] dealt with various aspects of shoot anatomy, and Srivastava [17] characterized gymnospermous features in its stem secondary phloem. It was not until 2001 that Carlquist [18] reported on root wood, with vessel elements, tracheids, and fiber-tracheids, and bark structure. He touched upon the cortex, which he stated had compound starch grains and some oil cells, and secondary phloem, which was comprised of sieve cells and parenchyma, but lacked sieve tube elements and sclerenchyma. 

Since nothing is known about *Austrobaileya* early root development and structure and RAM organization, our goals were to characterize them and the differentiation of primary tissues and secondary tissues during primary and early secondary root growth, and to evaluate if *Austrobaileya* shares more traits with other members of early-diverging lineages like *Amborella*, the closely related *Illicium* (Austrobaileyales), or the magnoliids. We were also interested to ascertain if they may still have some potentially new ancestral traits shared with extant and extinct gymnosperms (or other vascular plant lineages).

## 2. Materials and Methods

Material—About 30 adventitious and lateral roots of *Austrobaileya scandens* were collected from three 12-year old plants, which had been grown from seeds and cultivated in pots at the Weld Hill research building greenhouse in the Arnold Arboretum of Harvard University, Boston, Massachusetts. All roots were fixed in summer 2012 with 4% acrolein (Polysciences Inc., Warrington, PA, USA) in a modified PIPES buffer adjusted to pH 6.8 (50 mM PIPES and 1 mM MgSO_4_, both from BDH, London, UK; and 5 mM EGTA from Research Organics Inc., Cleveland, OH, USA) for 24 h. After rinsing with the same buffer, root material was dehydrated using an increasing ethanol dilution series and stored in 70% ethanol. 

Scanning Electron microscopy (SEM)—Five roots were rehydrated and treated with 2% Osmium tetroxide for 2 h before complete re-dehydration using an increasing ethanol dilution series up to 100% and a transfer in acetone overnight. All material was further processed with an AutoSAMDRI-815 automatic critical point dryer (Tousimis, Rockville, MD, US) and specimens were mounted on aluminum stubs before observations.

Histological sections—About half of the roots were sectioned by hand using double-edged Wilkinson razor feather blades, while 10 were further dehydrated using dilutions up to a 100% ethanol solution, infiltrated and embedded in methacrylate resins (JB-4, Polysciences, Warrington, PA, USA, or Kulzer’s Technovit 7100, Electron Microscopy Sciences, Hatfield, PA, USA), and cut in 4–10 µm thick serial sections using a rotary microtome (Thermo Fisher Scientific, Waltham, MA, USA) and disposable blades (Leica Biosystems, Wetzlar, Germany). All sections were stained generally following Jensen [19], either with saturated phloroglucinol-HCl for detection of lignin, berberine hemisulfate counterstained with 0.05% toluidine blue O (TBO) for detection of suberin, fluorol yellow for detection of lipids, calcofluor (i.e., Fluorescent Brightener 28) for detection of plant cell walls and fungal hyphae, Iodine Potassium (IKI) for detection of starch grains, or 0.05% TBO and 1.0% ruthenium red for general staining (all Sigma-Aldrich, Saint Louis, MO, USA). Resin sections were permanently mounted on glass slides using Citrisolv cleaning solution (Decon Labs, Inc., King of Prussia, PA, USA) and Permount (also Sigma-Aldrich) or Histomount (Agar Scientific, Stansted, United Kingdom) mounting media, and stored permanently at FU Berlin.

**Figure 1:**
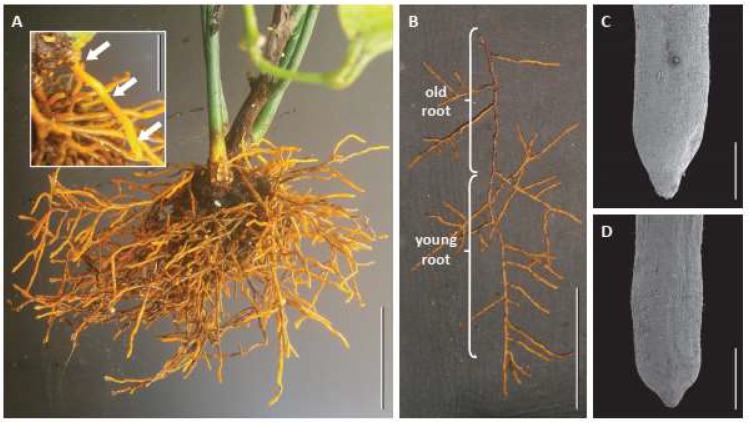
Root systems of *Austrobaileya scandens*. (**A**) root system with adventitious roots (inset: young adventitious root indicated by white arrows). (**B**) adventitious root bearing laterals, with old part covered by phellem, and young part still covered by epidermis. **C**,**D**: SEM pictures of roots with elongated (**C**) and truncated (**D**) tips, indicating continuous and arrested growth, respectively. Scale bars: **A**,**B** = 5 cm (1 cm in inset); **C**,**D** = 1 mm.

Image acquisition, processing and plates editing—Digital pictures of the roots in Figure 1 were taken using the camera of a Samsung Galaxy S7 cell phone (Samsung Inc., Maetan-dong, South Korea). In SEM images, acquired with an Hitachi100 Scanning Electron Microscope (Hitachi Ltd, Tokyo, Japan), a black background has been edited to provide better contrast around the smooth contour of the roots. All hand and microtome sections were studied using a Zeiss LSM700 confocal microscope mounted with a Zeiss MRc Axiocam camera, and using brightfield, Differential Interference Contrast (DIC), or epifluorescence illumination, and the 405 nm and 488 nm solid-state lasers (Carl Zeiss AG, Oberkochen, Germany). All pictures and plates were edited and arranged using Adobe Creative Suite (Adobe, San Jose, CA, USA).

## 3. Results 

### 3.1. Root System and Apex Structure

**Figure 2:**
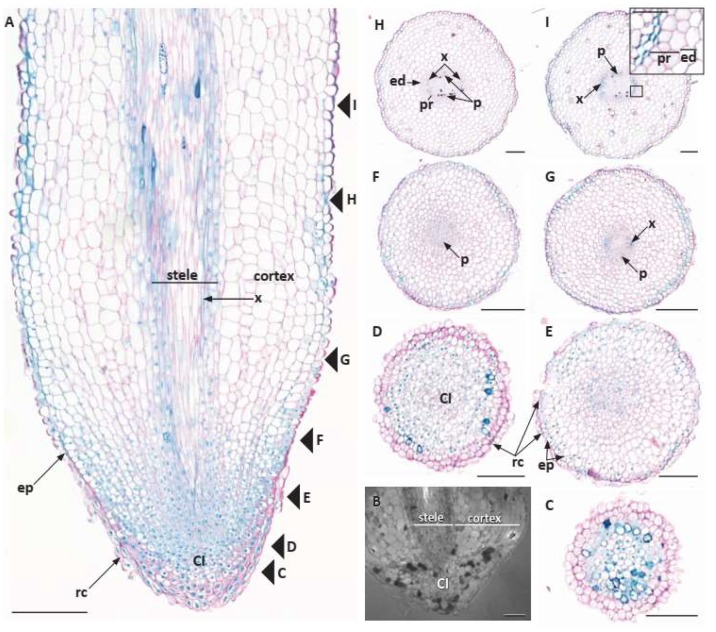
Root primary anatomy of *Austrobaileya scandens*. **A**: median longitudinal section of elongated root tip showing RAM with common initials and the major tissues of the central cylinder or stele, cortex, epidermis, and root cap. Arrowheads and letters designate positions of images in 5**C**–**I**. **B**: truncated root tip with arrested growth, common initials, and stele and cortex differentiated close to the RAM. **C**–**I**: transverse section series from root tip with root cap through common initials and tissues differentiating into epidermis, cortex with endodermis, and stele with pericycle, primary phloem, and primary xylem with diarch stele. **C**: < 0.2 mm, level of the root cap through the columella. **D**: at 0.2–0.4 mm, level of CI region in center. **E**: at 0.4–0.5 mm, level immediately proximal to CI with procambium region more densely stained in center. **F**: at 0.5–1 mm, level of first protophloem differentiation. **G**: at 1–1.5 mm, level of first xylem tracheid. **H**: at 1.5–3 mm, distinct diarch stele with multiple protophloem elements at p, pericycle and endodermis now evident. **I**: at 3–4 mm, level of diarch stele with multiple tracheids at x and protophloem at p, surrounded with 2–3 layers of pericycle and a layer endodermis (inset, with pericycle and endodermis). Abbreviations: CI: common initials; ed: endodermis; ep: epidermis; p: phloem; pr: pericycle; rc: root cap; x: xylem. Scale bars: **A** = 250 µm; **B**–**I** = 100 µm (50 μm in inset).

Multiple adventitious roots arose from basal regions of stems and yielded numerous lateral roots, forming a homogeneous orange and glabrous root system (Figure 1A). All adventitious roots and lateral roots were very similar (Figure 1B), and both had tips varying in shape from elongated to slightly truncated (Figure 1C,D and Figure 2A,B). 

In elongated root tips (Figure 2A), median longitudinal sections showed RAM with a root cap and its columella region not completely distinguishable from peripheral root cap tissues and common initials for the other associated tissues, protoderm, ground meristem, and procambium that were not traceable to the columellar region base (Figure 2A). While the procambium narrowed at its tip, it was not clearly separated by distinct cell walls or cell files from cells at the tip of the ground meristem or base of the columella region of the RAM, even though the procambium tended to stain more intensely than the surrounding ground meristem because its cells appeared to have a denser cytoplasm. The ground meristem was indistinguishable from the other common initials and, near its tip, did not have orderly files of cells emanating from its tip-most region. The same RAM structure was observed in truncated root tips (Figure 2B). 

In transverse sections, there was no distinct columella (Figure 2C), even though the procambium narrowed at its tip, its boundaries with the ground meristem could not be determined (Figure 2D). Beginning in the RAM (Figure 2D) and proceeding proximally, cell files could be seen longitudinally in the ground meristem (Figure 2A), but not radially, even though there were some extending from the periphery of the CI into nearby ground meristem (Figure 2A,E). However, the common initials in the RAM did not contribute to an orderly structure initiating the tissues, and a distinction between the procambium, ground meristem, and protoderm could not be made up to 1.0 mm behind the tip (Figure 2F). Between 0.5 and 1.5 mm behind the tip the first phloem elements appear (Figure 2F) followed by the differentiation of the first protoxylem tracheids (Figure 2G), and the premises of a stele surrounded by a fairly thick cortex (Figure 2F). 

**Figure 3:**
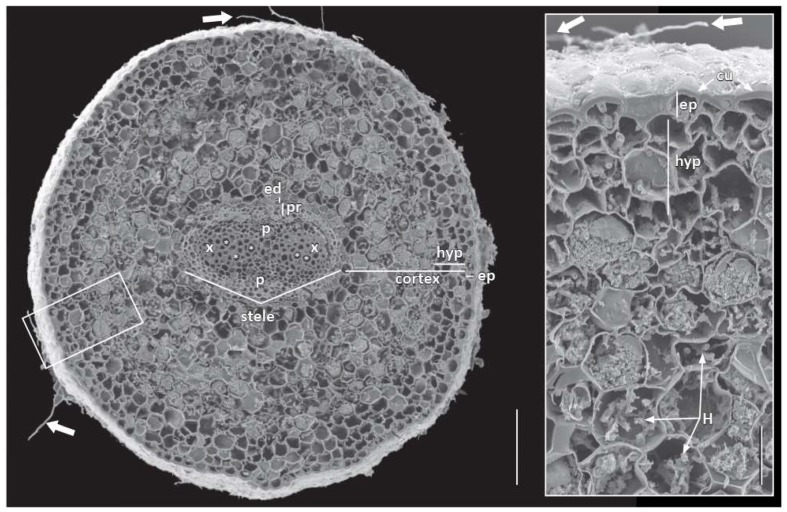
Transition zone between younger and older part of *Austrobaileya scandens* roots. Transverse SEM section and inset of root with glabrous epidermis covered with a thick cuticle and hyphae (white arrows), and surrounding a cortex with a three-cell-layer thick, multiseriate hypodermis surrounding a middle cortex colonized by arbuscular mycorrhizae. Oblong-shaped diarch stele, including tracheids and vessel elements (*), two protoxylem and protophloem poles, delimited by uniseriate endodermis and two–three cell-layers thick pericycle. Abbreviations: cu: cuticle; ed: endodermis; ep: epidermis; H: hyphae; hyp: hypodermis; p: phloem; pr: pericycle; x: xylem. Scale bar = 200 µm (50 µm in inset).

In the truncated tip cells broadened out immediately behind or proximal to the almost pyramidal-shaped root cap (Figure 1D and Figure 2B). Their ground meristem cells had swollen and the vascular tissues were differentiated almost down to the tip, indicating that they had stopped elongating. Such roots also appeared to be more likely to have a cortex packed with starch, sometimes with aseptate or septate inter- and intracellular hyphae, indicating that the roots may be mycorrhizal and/or harboring an endophytic fungus (Figure 3). 

### 3.2. Primary Root Growth and Tissue Differentiation

**Figure 4:**
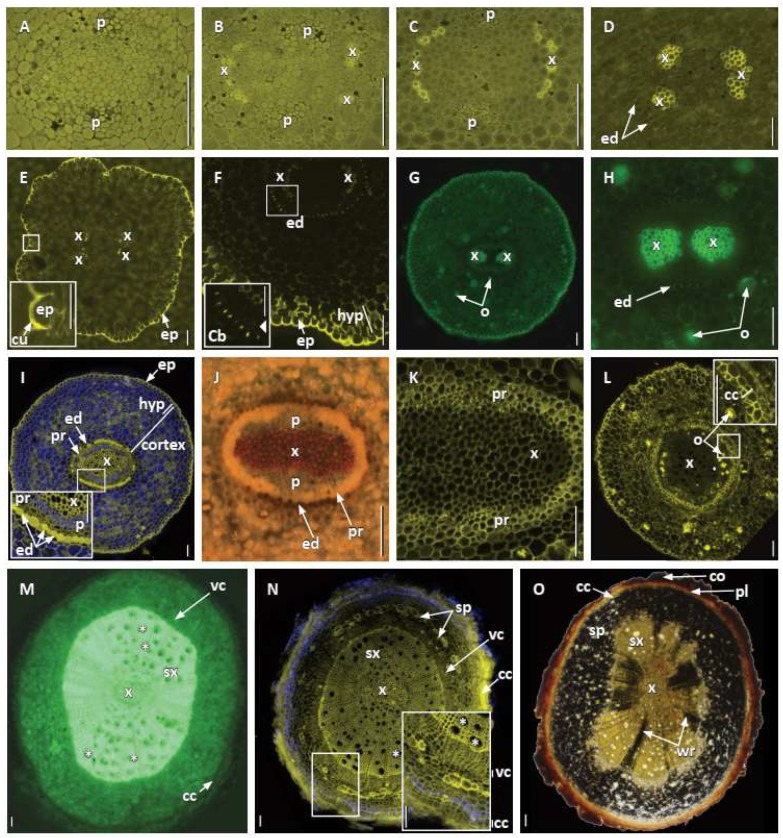
Primary and secondary tissue differentiation in *Austrobaileya scandens* roots. Transverse sections of different specimens imaged with laser (**A**–**F**,**I**,**K**,**L**,**N**), epifluorescence (**G**,**H**,**M**), and bright field (**J**,**O**) illumination, arranged in ontogenic series, with the youngest stage on the left of each row. **A**–**D:** microtome sections stained with berberine and TBO showing the successive differentiation of two phloem and two (more or less symmetrical) xylem poles, and endodermis. **A**: note protophloem elements. **B**: note separated protoxylem elements in one pole. **C**: note xylem poles with arc of tracheids in each pole. **D**: first endodermis with Casparian bands evident, one xylem pole with many tracheary elements and other xylem pole still with separate regions of tracheary cells. **E**–**H**: hand sections stained with fluorol yellow showing epidermis and endodermis, and diarch stele. **E**: diarch stele with separated protoxylem in each pole, epidermis with thick two-layered cuticle (inset). **F**: endodermis with Casparian bands (inset), three-layered exodermal hypodermis, epidermis with thick cuticle. **G**: diarch stele and oil cells. **H**: diarch stele with many tracheids and some vessel elements, endodermis, oil cells. **I**–**L**: hand sections stained with calcofluor (**I**) and phloroglucinol-HCl (**J**–**L**) showing transition from primary to secondary growth and successive differentiation of a multiseriate pericycle, with inner portion opposite protoxylem becoming vascular cambium and outer portion cork cambium, while the cortex and epidermis are still present. **I**: note metaxylem in center of stele and hyphae in inner cortex, and pericycle and endodermis in inset. **J**: full protoxylem and metaxylem, phloem, pericycle, and endodermis. **K**: primary xylem surrounded by wide pericycle. **L**: early stage of cork cambium and phellem development, oil cells still evident. **M**: hand section stained with berberine showing primary xylem in center, vessels (some marked by asterisks *), few vessels in secondary xylem outside protoxylem poles in early secondary growth. **N**: hand section stained with calcofluor showing more secondary growth, vessels (*) mostly throughout secondary xylem, secondary phloem fibers under arrows at sp, cork cambium with phellem (inset). **O**: hand section stained with IKI showing primary and secondary xylem with wood rays with starch grains, secondary phloem, cork cambium, phellem, and remnant cortex. Abbreviations: Cb: Casparian bands; cc: cork cambium; co: cortex; cu: cuticle; ed: endodermis; ep: epidermis; hyp: hypodermis; o: oil cells; p: phloem; pl: phellem; pr: pericycle; sp; secondary phloem; sx: secondary xylem; vc: vascular cambium; wr, wood rays; x: primary xylem. Scale bars = 100 µm (50 µm in insets).

The first protophloem cells typically differentiated and matured prior to protoxylem tracheid differentiation and maturation (Figure 2F and Figure 4A). More protophloem elements were produced just laterally to the first element and the first protoxylem cells became prominent across each broad xylem pole (Figure 2G and Figure 4B,C). Diarchy was characteristic (Figure 2F–I and Figure 4B–H). Sometimes, there appeared to be asymmetric xylem patterns with one xylem pole exhibiting separated protoxylem cells or clusters of cells (Figure 4B,D,E), or even both xylem poles with slightly separate protoxylem cells in each pole, in which the protoxylem tracheids had not differentiated and matured uniformly across a pole (Figure 4C–E). However, the protoxylem production revealed that there were typically still only two xylem poles with numerous tracheids in each (Figure 4D,F–H) and a vessel element or two in a pole (Figure 4H). Primary xylem gradually filled in the center of the vascular cylinder (Figure 3 and Figure 4I,J) and vessel elements could be identified (e.g., Figure 3 and Figure 4H,I). While we did not specifically test for phloem elements, we could identify them by cell position and shape (e.g., Figure 2H,I and Figure 4A–C).

The pericycle early in primary growth became multiple cell layers (Figure 4F,I–K), and although it remained unstained with phloroglucinol-HCl (Figure 4J), it can be viewed in SEM (Figure 3). At first, it was also difficult to discern a discrete endodermal layer (Figure 2E,G and Figure 4). However, the cortex became delimited by an endodermis with distinct Casparian bands, which covered over half of the radial walls (Figure 4D,F,H,I). Suberin lamellae were then produced in most endodermal cells opposite the two phloem poles of the stele, leaving all endodermis cells opposite the large protoxylem poles as passage cells (Figure 4H,I). In the endodermis most cells developed suberin lamellae (Figure 4I).

The mid cortex comprised many cells that varied depending on the sizes of adventitious and lateral roots, and there were oil cells (Figure 4G,H), small air spaces (Figure 4I), and many cells that contained multiple, compound starch grains (Figure 3). A hypodermis generally differentiated into a three-cell-layer thick exodermis with fewer cytoplasmic contents and with Casparian bands developing on its radial (and transverse) cell walls (Figure 3 and Figure 4E,F,I,L). The exodermis also had thin suberin lamellae, which accounted for the fluorescence on the tangential cell walls (Figure 4F,I), in addition to the radial and transverse cell walls. There were also mycorrhizal hyphae in the mid cortex cells of many roots (Figure 3). 

Outer cell walls in the root epidermis had a very thick cuticle of two distinct layers (Figure 3 and Figure 4E,G,I,L), even down to the proximity of the root tip under peripheral root cap cells (Figure 2A,E–H). There were neither trichoblasts nor root hairs, but mycorrhizae could be observed on the epidermis (Figure 3).

### 3.3. Secondary Root Growth and Tissue Differentiation 

**Figure 5:**
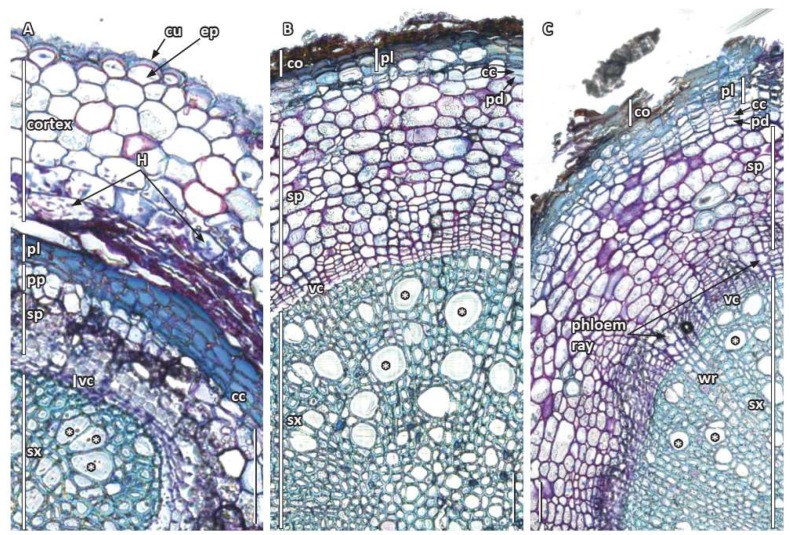
Secondary tissue differentiation in *Austrobaileya scandens* roots. Transverse sections of one specimen arranged in ontogenic series, with the youngest stage on the left and transition from primary to secondary growth (**A**), to those of early (**B**) and later (**C**) secondary growth, stained with TBO and ruthenium red. **A**: beginning of secondary growth, vascular cambium, early secondary phloem, remnant primary phloem, cork cambium, and phellem. Note the innermost part of the cortex with hyphae is crushed, while the outermost cortex and epidermis with a thick two-layered cuticle are still present. **B**: secondary xylem with vessel elements (*), vascular cambium, secondary phloem, layer of phelloderm at arrow, cork cambium, phellem, and remnant cortex. **C**: secondary growth with primary and secondary phloem and xylem (vessels at *), wood and phloem rays, phelloderm, cork cambium and multiseriate phellem, and remnant cortex. Abbreviations: cc: cork cambium; co: remnant cortex; cu: cuticle; ep: epidermis; H: hyphae; pd: phelloderm; pl: phellem; pp: primary phloem; sp: secondary phloem; sx: secondary xylem; vc: vascular cambium; wr: wood ray;. Scale bars = 100 µm.

As noted above, the pericycle stained and photographed differently from the internal procambium, primary xylem and phloem (Figure 3 and Figure 4I–K). Procambial cells between primary xylem and phloem and inner pericycle cells over the xylem poles became vascular cambium (Figure 4I–K). Secondary xylem was somewhat asymmetrically produced with vessel elements being formed outside protophloem (Figure 4M–O). The cork cambium or phellogen differentiated from the outer layers of redivided pericycle (Figure 4I–L) and started to produce the first phellem cells under the endodermis and just outside phloem (Figure 4L,inset and Figure 5). As the cork cambium proliferated (Figure 4L,N,O and Figure 5A), multiple layers of phelloderm were produced to the interior of the phellogen (Figure 5B,C). While the root expanded secondarily, more vessel elements were produced along with tracheids, parenchyma, and rays, and eventually became distributed throughout the secondary xylem (Figure 4N and Figure 5B,C), as first described by Carlquist [18]. Secondary phloem (Figure 4N,O and Figure 5B), with sieve cells, parenchyma and wider rays with starch (Figure 4O and Figure 5C), were also as described in more detail by Carlquist [18]. We also detected phloem fibers (Figure 4L,N and Figure 5C) and noted that starch accumulated in parenchyma cells and phloem ray cells. The cortex, with its starch, oil cells, and mycorrhizae, and the epidermis remained around the root for a considerable distance behind the root tip (Figure 4N,O and Figure 5A,B), but eventually are pushed outward, disintegrated, and sloughed off (Figure 5).

## 4. Discussion

We started this project because we were interested in possible pleisiomorphic characteristics in this representative of the ANA grade and basal angiosperms after the broad comparative studies of Clowes [20] with 157 angiosperm species (including five Nymphaeales [Nymphaeaceae: *Nymphaea*], two Austrobaileyales [Schisandraceae: *Schisandra*, *Kadsura*], 15 magnoliids, 65 monocots, and 70 eudicots), Groot et al. [21] with 66 species (including two Nymphaeales [Nymphaeaceae: *Nymphaea*], three magnoliids, and 61 eudicots), and Heimsch and Seago [4] with 425 species (including *Amborella* of the Amborellales, three Nymphaeales [Nymphaeaceae: *Nuphar* and *Nymphaea*], two Austrobaileyales [Schisandraceae: *Illicium*, *Schisandra*], eight magnoliids, 45 monocots, and 368 eudicots). 

RAMs were consistently comprised of common initials in *A. scandens*, whereas considerable variability was reported in roots of *Amborella* and in *Illicium* (Schisandraceae), the only other member of the Austrobaileyales that has been investigated to date [4]. Nevertheless, *Amborella* and *Illicium* share some characteristics with *Austrobaileya* in that some of their roots with open RAMs occasionally have a stronger resemblance to coniferous gymnosperm RAMs [6,8] than to other gymnosperms [7]. Manifestations of the RAM with common initials in gymnosperms and some early-diverging lineages angiosperms, including members of the ANA grade and magnoliids, which also exhibit RAM common initials, must reflect the ancestral condition of this structural feature of roots and may be pleisiomorphic for seed plants as a whole [4,22].

In many respects, *A. scandens* roots also exhibited primary growth generally similar to *Amborella* and *Illicium* roots [5], although *A. scandens*, along with *Illicium* but unlike *Amborella*, had tracheids and vessel elements [18,23,24]. In *A. scandens,* primary phloem with sieve cells was more prominent, and there was no triarchy or tetrarchy as observed in some *Amborella* or *Illicium* roots, respectively. Yet, all exhibit a cortex illustrative of differentiation from common initials and endodermis and exodermis [5]. Although it has vessel elements, unlike *Amborella*, the presence of common initials in the RAM and of phloem with sieve cells [18] provides evidence of a pleisiomorphic state for *A. scandens*. 

Early secondary growth in *A. scandens* had vessel elements somewhat restricted to regions opposite the broad swath of the phloem poles [18], whereas in *I. floridanum,* solitary vessel elements were scattered throughout [5]. In some ways, roots of *A. scandens* were similar to the roots of *Amborella trichopoda,* with a simple diarchy in which some protoxylem in each pole is slightly separated from other protoxylem of the same pole, probably by means of delayed maturation. Phloem was characterized by sieve cells and not sieve tube elements as found in most angiosperms, and this trait in *A. scandens* was similar to the phloem in *Amborella* [18]. Additionally, the endodermis had similar Casparian bands and early production of suberin lamellae opposite broad phloem poles [5]. Carlquist [18] had previously identified tracheids and vessel elements in these roots, and as Bailey and Swamy [9] had positively identified sieve cells. While Carlquist [18] did not describe any sclerenchymatous cells in root secondary phloem in *A. scandens*, we found scattered fibrous cells in the secondary phloem, similar to those found in stems. Similar to our findings, Carlquist [18] demonstrated a multiple-layered phelloderm under the phellogen and phellem.

Another fascinating feature of adventitious roots in *A. scandens* was the presence and thickness of a cuticle over the outer walls of epidermis and even its precursor protoderm and total absence of root hairs. The thick cuticle, as well as the presence of mycorrhizae, probably precluded the production of root hairs [20,25] and maybe a distally produced cuticle in roots contributed to or was a result of slow growth. However, that there were no trichoblasts does not mean that root hairs cannot be produced, like in roots of magnoliids where as Baylis [26] noted, they often tend to be absent or, if present, appear to be slow-growing or stunted. Our study also revealed that starch grains accumulated during early primary root growth in the cortex and in secondary phloem, including phloem rays of older roots. Furthermore, since the cells of the middle cortex were pushed out beyond the secondary growth periderm, they in effect deposited outside the root any remnant starch, oil, and mycorrhizae still in those cells. While the presence of starch in roots of *A. scandens* had already been reported in the field [14], the presence of mycorrhizae was reported and illustrated here for the first time and needs to be confirmed in its natural habitat. Oil cells were found in the cortex of young roots, and Srivastava [17] and Carlquist [18] reported them in periderm of older roots. Oil droplets were also recently documented in the starchy endosperm of the seeds of *A. scandens* [27], which are by far the largest of the ANA grade and among the largest of magnoliids. Their composition and function remain unclear, although the presence of ethereal oils has been reported earlier in *Illicium* and many other magnoliids, such as Aristolochiaceace or Piperaceae, and were easily perceived when crushing the roots of *A. scandens*. 

Two final points should be made. The distally thick cuticle may also prevent organisms in the rhizosphere from access to substances in the root during early root growth, and one of the possible outcomes of the disintegration of cortical cells with their contents of starch, oil, and mycorrhizae could be their availability to microorganisms in the soil. Lastly, as per Baylis [14,25], maybe this developmental trait of early angiosperms facilitated their survival and evolution.

## 5. Conclusions and Perspectives

We suggest that the characteristics we report for *A. scandens* are consistent with shared plesiomorphic traits reported for other members of the ANA grade of basal angiosperms and that these anatomical traits should aid scholars to better understand the phylogenetic and evolutionary relations of early-diverging and basal lineages of extant and extinct angiosperms.

Previous studies also identify different types of RAM, from open meristem with CI as found in early-diverging and basal angiosperms, to main types of open and closed meristems in monocots and eudicots. However, these types can sometimes shift during the ontogeny of a plant, as suggested by the co-occurrence of open RAM with CI and different types of closed meristems in *Amborella* and *Illicium*, and their evolutionary relationships and developmental transitions which may bind them remain to be resolved. Given the rather stable and robust current phylogeny of angiosperms, a re-evaluation of RAM types and their correlation with other root traits could contribute to reconstruct the root traits of the angiosperm common ancestor and shed a new light on their ecological origin and rapid subsequent radiation.
